# Evidence of Statistical Inconsistency of Phylogenetic Methods in the Presence of Multiple Sequence Alignment Uncertainty

**DOI:** 10.1093/gbe/evv127

**Published:** 2015-07-01

**Authors:** A.S. Md Mukarram Hossain, Benjamin P. Blackburne, Abhijeet Shah, Simon Whelan

**Affiliations:** ^1^Faculty of Life Sciences, University of Manchester, United Kingdom; ^2^Evolutionary Biology, Evolutionary Biology Centre, Uppsala University, Sweden

**Keywords:** multiple sequence alignment, phylogenetic inference, maximum likelihood, sequence divergence, tree inference

## Abstract

Evolutionary studies usually use a two-step process to investigate sequence data. Step one estimates a multiple sequence alignment (MSA) and step two applies phylogenetic methods to ask evolutionary questions of that MSA. Modern phylogenetic methods infer evolutionary parameters using maximum likelihood or Bayesian inference, mediated by a probabilistic substitution model that describes sequence change over a tree. The statistical properties of these methods mean that more data directly translates to an increased confidence in downstream results, providing the substitution model is adequate and the MSA is correct. Many studies have investigated the robustness of phylogenetic methods in the presence of substitution model misspecification, but few have examined the statistical properties of those methods when the MSA is unknown. This simulation study examines the statistical properties of the complete two-step process when inferring sequence divergence and the phylogenetic tree topology. Both nucleotide and amino acid analyses are negatively affected by the alignment step, both through inaccurate guide tree estimates and through overfitting to that guide tree. For many alignment tools these effects become more pronounced when additional sequences are added to the analysis. Nucleotide sequences are particularly susceptible, with MSA errors leading to statistical support for long-branch attraction artifacts, which are usually associated with gross substitution model misspecification. Amino acid MSAs are more robust, but do tend to arbitrarily resolve multifurcations in favor of the guide tree. No inference strategies produce consistently accurate estimates of divergence between sequences, although amino acid MSAs are again more accurate than their nucleotide counterparts. We conclude with some practical suggestions about how to limit the effect of MSA uncertainty on evolutionary inference.

## Introduction

Studies of molecular evolution typically wish to infer the patterns by which sequences change over time, using those changes to reveal evolutionary relationships (Felsenstein 2003), patterns of selection acting upon genes (Yang 2006), or other biologically informative quantities (Galtier and Gouy 1998; Whelan et al. 2011; Liberles et al. 2012). The inferential process is typically split into two distinct steps: the first is to infer the sitewise homologies among the sequences through multiple sequence alignment (MSA; see Anisimova et al. 2010 for discussion); and the second is to use those sitewise homologies to make inferences about the evolutionary process through phylogenetic inference. Early in the development of MSA it became clear that the MSA and evolutionary inference steps are integral to one another (Sankoff and Kruskal 1983), but the computational convenience of separating the two steps has led to the development of MSA methods (MSAMs) and methods of phylogenetic inference becoming increasingly separated from one another, further consolidating this two-step inferential process. This study will investigate how these two steps interact by examining how the MSA step affects the well-characterized statistical properties of the evolutionary inference step. We begin by highlighting the core developments in MSAMs and phylogenetic inference.

MSAM developers have tended to concentrate on improving their software benchmark performance on structure-based MSAs (Blackshields et al. 2006), which capture functional or structural similarity between amino acids in proteins, but may or may not accurately reflect the sitewise homologies required for evolutionary studies. Since the development of the early progressive MSAMs ALIGN and CLUSTAL (Dayhoff and Schwartz 1978; Thompson et al. 1994), there have been many key algorithmic developments, which have lead to improvements in MSAM performance assessed via structural benchmarks. These improvements include: iterative progressive alignment, where the guide tree and subsets of the MSA are iteratively updated (Edgar 2004); consistency alignment, where local patterns of conservation and change are integrated into an MSAM (Notredame et al. 2000; Do et al. 2005); and hybrid MSAMs that attempt to use the best from all approaches (Wallace et al. 2006; Katoh and Standley 2013). More recently, some of the principles of molecular evolution have been applied to MSA in Prank, an MSAM that includes the explicit modeling of substitutions and the phylogenetically meaningful placement of insertions and deletions during a sequences history (Löytynoja and Goldman 2008). Benchmarking studies have shown that Prank performs rather well on data simulated from a phylogenetic model, but notably less well relative to other MSAMs on structurally derived benchmarks (Blackburne and Whelan 2012).

In contrast, researchers developing phylogenetic inference methods have mostly ignored any uncertainty associated with the MSA step, and instead have concentrated on developing methods that assume a known MSA. Many of the key developments in phylogenetic inference methodology have been linked to the statistical properties of those methods (Felsenstein 2003). Statistical methods, such as maximum likelihood (ML) and Bayesian inference, have been shown to be statistically consistent (Rogers 1997), which means they tend toward the correct answer as more data are added to an analysis conditional on the substitution model being correct. The consistency property is one of the key reasons for the adoption of statistical methods over maximum parsimony (MP), which was demonstrated to be inconsistent and susceptible to long-branch attraction (LBA) artifacts (Felsenstein 1978). Other key methodological advances in phylogenetic inference have all assumed that the patterns of substitution observed in sequences are reflective of a biologically informative evolutionary signal, such as natural selection or speciation, and independent of assumptions made during MSA (Felsenstein 2003; Kosiol et al. 2006; Yang 2006; Liberles et al. 2012).

Although methods for MSA and phylogenetic inference have mostly been developed independently of one another, there have been a number of studies that have either attempted to link the two steps together or demonstrated the effect of MSA on downstream evolutionary inference. The former has concentrated on either explicitly modeling substitution, insertion, and deletion through variants of the TKF model (Thorne et al. 1991, 1992; Redelings and Suchard 2005; Arunapuram et al. 2013), or iteratively refining MSA and phylogenetic tree estimates (Liu et al. 2009, 2012). Both of these approaches offer alternatives to the dogma of the two-step process, but are computationally demanding and, at present, too slow for large-scale genomic studies. The links between MSA and phylogenetic inference were established by early empirical studies showing that MSA could affect tree estimates and bootstrap support (Morrison and Ellis 1997). More recent empirical studies have shown that variation in the output of MSAMs leads to differences in estimates of phylogenetic trees and the detection of molecular adaptation (Wong et al. 2008; Markova-Raina and Petrov 2011; Blackburne and Whelan 2013). These have been supported by simulation studies, showing that choice of MSAM has an effect on MSAs (Blackburne and Whelan 2012), phylogenetic tree estimates (Liu et al. 2009), and the detection of molecular adaptation (Fletcher and Yang 2010). Blackburne and Whelan (2013) suggested that the greatest difference in evolutionary inference was between MSAMs based on evolutionary principles (evolutionary-MSAMs) and those based on more orthodox similarity-based approaches (similarity-MSAMs).

Although these studies demonstrate a link between MSA and the accuracy of genomic inference, they have not addressed the general statistical properties of phylogenetic inference under an unknown MSA. Here, we use a simulation study to examine whether phylogenetic inference can recover accurate divergence and tree estimates under the correct substitution model without assuming a known MSA. If phylogenetic inference is statistically consistent in the presence of MSA uncertainty we expect two key properties to hold when the simulation and inference model are the same. First, MSA will introduce uncertainty of parameter estimates in phylogenetic inference, but it will not introduce a bias to estimates relative to estimates made from the true MSA. Second, these estimates will tend asymptotically to the correct answer as more data are added. Here, we provide evidence that uncertainty and inaccuracy in MSA can bias estimates of sequence divergence, both in terms of the individual branch lengths on a known tree and the total tree length (sum of branch lengths). We then proceed to demonstrate that MSA leads to inaccurate phylogenetic tree estimates, first by showing that MSA leads to arbitrary and statistically supported resolution of multifurcations, and then by providing evidence for a “Felsenstein zone” where LBA artifacts occur for some types of data and some MSAMs. We conclude with some practical precautions that researchers can take to ensure their results are as accurate as possible in the face of evidence of systematic bias caused by MSA.

## Materials and Methods

### Specifying the Simulation Model

Simulation of sequence evolution under nucleotide and amino acid sequences was conducted using INDELible. Nucleotide sequences were simulated under the general time reversible model (GTR) with four categories of Γ-distributed rates across sites. Our parameterization of the rate matrix is inspired by the mammalian genes analyzed by Arbiza et al. (2011), with exchangeability parameters $r_{AC}=0.30$, $r_{AG}=1.0$, $r_{AT}=0.20$, $r_{CG}=0.25$, $r_{CT}=1.39$, $r_{GT}=0.22$; and nucleotide frequencies $\pi_{A}=0.25$, $\pi_{C}=0.26$, $\pi_{G}=0.27$, $\pi_{T}=0.22$. Amino acid sequences were simulated under Whelan and Goldman (WAG) with four categories of Γ-distributed rates across sites. For both state-spaces we chose α=1.8 for the parameter of the Γ-distribution, based on a preliminary study examining parameters inferred from PANDIT families (Whelan et al. 2003, 2006). Insertion and deletion is described by a reversible process, with insertions and deletions occurring 0.05 times the rate of substitutions, which is inspired from estimates obtained from Cooper et al. (2004). Following the default values in INDELible we use a power law distribution to describe indel length, with an α value of 1.7. We impose a maximum indel length of 20 (60) characters for amino acids (nucleotides). This length is relatively short, but is chosen to ensure direct and clear pairwise homology between even the more divergent sequences in our simulations. The length of the root sequence is inspired by the average length of sequence of a subset of PANDIT families. Unless specified otherwise the length of the root sequences are taken of the tree are 408 amino acids and (3 × 408=) 1,224 nt.

**Fig. 1.—:**
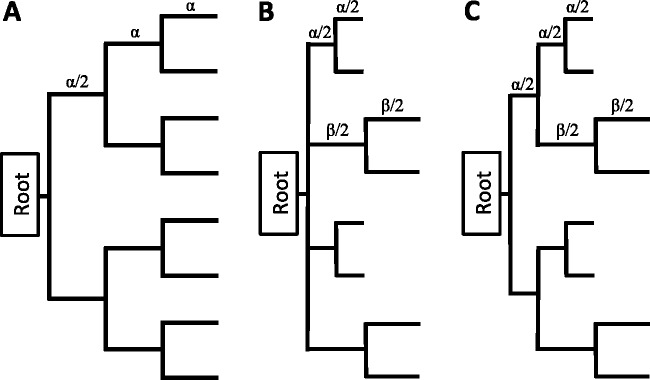
The evolutionary trees used for simulation in this study. Tree *A* is a balanced tree, used to investigate branch length estimates. All branch lengths are equal, with the whole tree scaled to obtained different tree lengths. Note the definitions of sets of branches used later in figure 3. Tree *B* is an eight-taxa balanced tree with a multifurcation at the root, used to investigate how MSAMs resolve multifurcations. Tree *C* is an eight taxa “Felsenstein-style” tree with two long and two short subtrees, used to investigate LBA artifacts.

The tree topologies used for simulation are shown in figure 1are intended to represent: A) a balanced tree with a confident topology, which we use to study how estimates of divergence vary by aligner; B) a single deep degree 4 multifurcation (polytomy), which we use to examine whether aligners systematically affect tree estimates; and C) a Felsenstein-zone style tree, which we use to examine whether aligners can induce LBA artifacts in phylogenetic analyses.

### MSA and Quantifying MSA Error

MSA was conducted using 11 popular alignment algorithms. We investigate the progressive aligners ClustalW (Thompson et al. 1994), Clustal Omega (Sievers et al. 2011), MUSCLE (Edgar 2004), and MAFFT FFT-NS-2, which is the progressive algorithm of the MAFFT package (Katoh and Standley 2013); the consistency aligners T-Coffee (Notredame et al. 2000), ProbCons (Do et al. 2005), FSA (Bradley et al. 2009), and MAFFT L-INS-i (Katoh and Standley 2013); and the phylogenetically aware aligner Prank (Löytynoja and Goldman 2008). In addition to these standard algorithms we also examine SATé, which attempts to improve MSA estimates by using a divide-and-conquer approach to MSA based on MAFFT and the phylogenetic tree inference program RAxML to update the guide tree. All aligners are run using default settings. Modifying these settings may yield better or worse estimates of evolutionary parameters, but do not reflect how the vast majority of researchers use these programs. Comparisons between resultant MSAs are conducted using MetAl (Blackburne and Whelan 2012) under the $d_{\text{evol}}$ metric or FastSP to obtain true positive and false negative scores (Mirarab and Warnow 2011).

### Phylogenetic Inference

The majority of phylogenetic inferences are conducted using the PAML package (Yang 2007b) under a small number of selected trees. The choice of the PAML package is to obtain high accuracy of ML branch length and topology estimates, which ensures the effects we study are the product of ML inference and not a quirk of a heuristic and fast tree-search program. To analyze nucleotide sequences we use the GTR model with four categories of Γ-distributed rates across sites, implemented in the baseml program. For amino acid sequences we use WAG with four categories of Γ-distributed rates across sites, implemented in codeml. An appropriate set of phylogenetic trees is specified a priori depending on what is being investigated. Otherwise all parameters are estimated from the data using ML, with the exception of nucleotide and amino acid frequencies, which are estimated using their observed counts. Inference of sequence divergence, in terms of total tree length and individual branch lengths, is performed using ML on the same tree that was used to generate the data. Tree inference on data generated by the multifurcation presented in figure 1*B* is done by estimating ML for each of the three possible resolutions of that multifurcation and choosing the best. In cases where several trees have the same ML (tolerance 1.0E-2 log-likelihood units) each tree was weighted equally so the total contribution of trees from that simulated data sets was one. SH-tests (Shimodaira and Hasegawa 1999) on the resultant trees were conducted using the native code in PAML. Trees were rejected by the SH-test at α=0.05 and the panels in figure 7 simply show the frequency that each tree was rejected across all simulations. Note that the ML tree for each simulation cannot be rejected. Tree inference on data generated from the Felsenstein-zone tree (fig. 1*C*) is done in a similar manner to that for the multifurcation, although we do not perform SH-tests.

## Results and Discussion

This study investigates the effect of MSA on phylogenetic inference through data simulated by INDELible (Fletcher and Yang 2009). We examine amino acid sequence data from WAG+F+Γ (Whelan and Goldman 2001) and nucleotide sequence data from GTR+Γ (Yang 1994), parameters inspired by studies based on large repositories of genomic data, in an effort to ensure they reflect some of the properties of real sequences (Whelan et al. 2006; Arbiza et al. 2011). We also include an insertion and deletion process, with estimates taken from mammalian genomic data (Cooper et al. 2004). Inference of phylogenetic models and trees are performed using the two-step process of MSA followed by ML inference, using the same substitution models used to simulate data, but treating gaps as missing data in line with standard phylogenetic methodology. Under these conditions proofs of statistical consistency show that given the true MSA and enough data all phylogenetic parameters can be estimated accurately in an unbiased manner. We examine the effect of using MSAMs on the inference of three quantities important in evolutionary biology: 1) the divergence between sequences; 2) the phylogenetic tree estimate; and 3) the statistical confidence in that tree estimate. Note that the simulations in our study use eight or more taxa to ensure MSA is performed across multiple internal nodes. These internal alignments are often where program specific heuristics are used, such as phylogenetic gap placement by Prank or profile-profile alignment in other MSAMs, and therefore represent the major differences between MSAMs.

### MSA Error and the Inference of Sequence Divergence

We simulate sequences on an eight species balanced tree (fig. 1*A*), where all branches are of equal length and the simulation starts from a root set halfway along the deepest branch. The tree-shape used in this study design is intended to be amenable to MSA because the guide tree should be relatively simple to infer and the sequences are evenly spaced on that tree. For divergent sequences the guide-tree estimates can become unreliable and the effect this has on the MSA and downstream analysis is discussed later.

### Inferring Total Sequence Divergence from an MSA

**Fig. 2.—:**
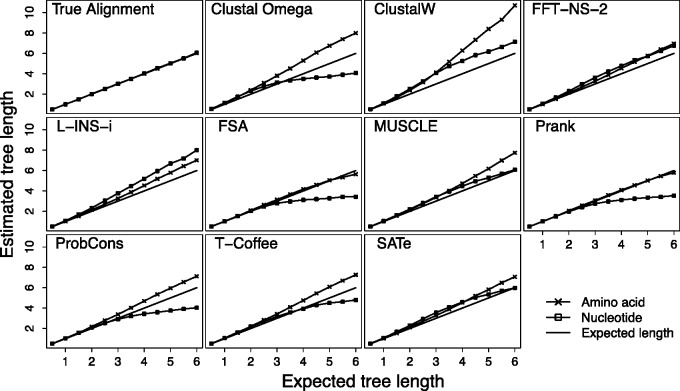
True (simulated) tree lengths and the median inferred tree lengths inferred from amino acid and nucleotide sequences. Deviation from the line *x* = *y* suggests estimates obtained from an MSA are biased.

The first aspect of sequence divergence, we examine is the ability for phylogenetic methods to correctly infer the total number of substitutions occurring between a set of sequences given a known phylogeny. Figure 2 shows the simulated (true) and inferred total tree length (the total sum of branch lengths) for amino acid and nucleotide sequences under a wide range of different MSAMs, each with its own panel. In all plots the solid line is x=y, represents what we should expect if the inferential procedure is working correctly. In line with our expectations based on statistical consistency, if we use the true alignment given by the simulation program we are able to obtain unbiased estimates of the tree length for both nucleotide and amino acid sequences.

There are several general points to take from our results. The divergence estimates obtained from most MSAMs show substantial differences in their accuracy when using nucleotide and amino acid sequences. All MSAMs—with the exception of MAFFT—tend to produce noticeably lower estimates from nucleotide sequences than from amino acid sequences. Of these MSAMs, most give underestimates of divergence from nucleotide sequence and relatively accurate to overestimates of divergence from amino acid sequences. The underestimation of divergence from nucleotide sequences is most severe with FSA and Prank, although both of these methods provide the most accurate divergence estimates from amino acid sequences. SATé performs similarly to other programs for amino acid sequences, whereas for nucleotide sequences the performance is similar to MUSCLE, with increasing overestimation for moderate divergences that gradually decreases as divergence increases.

### Patterns of Divergence Estimate Error across a Tree Topology

**Fig. 3.—:**
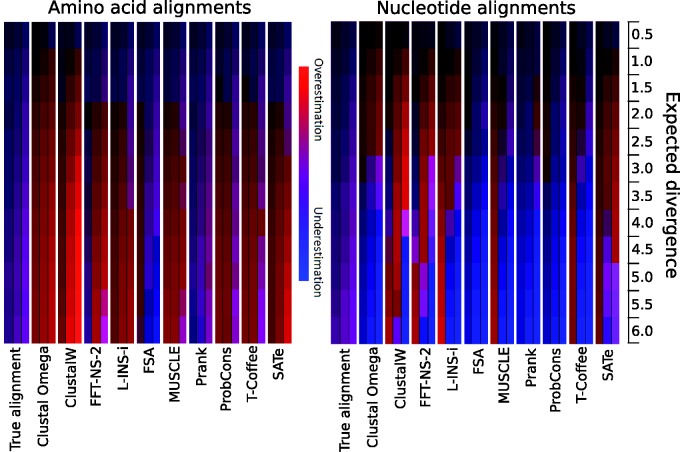
Summary of errors in branch length estimates across the tree for amino acid and nucleotide sequences. For each MSAM the left, middle, and right column corresponds to the external, middle, and root branches, respectively (see fig. 1*A*). The intensity of blue represents the difference between the lower quartile of estimates and the expected (simulated) branch length, whereas the intensity of red represents the difference between the upper quartile of estimates and the expected (simulated) branch length. A tendency toward blue indicates underestimates; red indicates overestimates, and the combination of both red and blue (violet) indicates unbiased estimates. The overall intensity of the color represents the variance of those estimates.

The previous section only investigates the overall accuracy of divergence estimates and implicitly assumes that they are evenly distributed through the tree structure. If errors in estimates of divergence between species were consistent between lineages then these errors would be of limited concern in evolutionary studies because they could be accounted for by a simple linear scaling factor. In contrast, if patterns of overestimation/underestimation are differentially distributed across the tree topology then it is likely to be problematic for phylogenetic analyses. The heat-map shown in figure 3 summarizes which branches on the phylogenetic tree errors are made when estimating divergence under the full range of conditions examined above.

For each aligner the left, middle, and right columns show a heat map indicating the type and intensity of error made for the external, middle, and root branches, respectively (see fig. 1*A*). The frequency with which MSAs lead to overestimation of branch lengths is shown by the intensity of red in the figure, whereas the frequency with which MSAs lead to underestimation is shown by the intensity of blue. In cases where an estimate has the correct mean and there is equal underestimation and overestimation, then the intensity of violet indicates the overall variance of the estimator, with the secondary color demonstrating an equal contribution of overestimates (red) and underestimates (blue). For example, theory predicts that the true MSA for both amino acids and nucleotides should provide accurate estimates of branch lengths, but their variance differs through the tree. This is reflected in the true alignment for both nucleotide and amino acid sequences with the variation in the intensity of violet for external, middle, and root branches. As expected, external branches have lowest variance and root branches the highest variance.

For the estimates obtained from the output of MSAMs, divergence estimates from both amino acid nucleotide sequences show that patterns of over- and underestimation are more variable, with different MSAMs and branch divergences suffering from over- and underestimation. This observation supports the hypothesis that MSAM error tends to be nonrandomly distributed through a tree topology. For amino acid sequences the patterns of overestimation evident from most aligners are nonrandomly distributed across the tree. The performance of FSA and Prank is of particular interest because when those MSAMs are applied to amino acid sequences they tend to produce MSAs that give a good overall estimate of divergence (see fig. 2), but this apparent accuracy results from a compound effect of two different types of error. MSAs from FSA tend to overestimate the external branches of a tree, but provide underestimates of the middle and root branches. In contrast, MSAs from Prank tend to lead to underestimates of external branches and (mild) overestimates of root branches.

Results obtained from MSAs from nucleotide sequences are more variable. Most MSAMs tend to give progressively worse underestimates of the root branch divergence as tree length increases. ClustalW is one notable exception since it tends to overestimates of the root branch divergence for moderate tree lengths, but underestimates it for longer tree lengths. A second is SATé, which mildly overestimates the root branch at low divergences, but becomes more accurate at higher divergences. The majority of other MSAMs underestimate the middle and there is a roughly even mix of over- and underestimation of external branches.

### The Relationship between MSA Error and Inference of Sequence Divergence

**Fig. 4.—:**
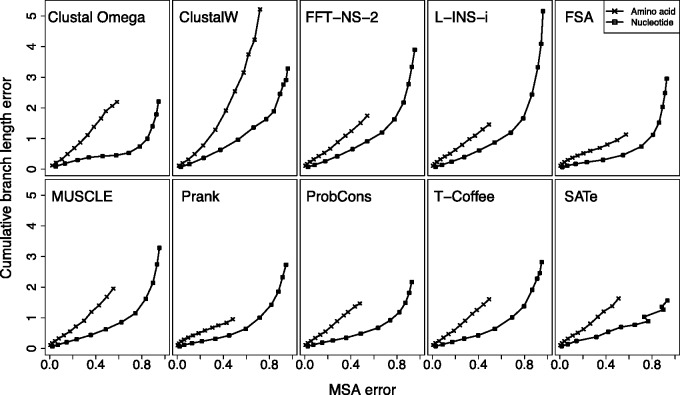
The relationship between MSA error, quantified by the *d*_evol_ distance from the true MSA, and cumulative branch length errors for amino acid and nucleotide sequences.

The above results demonstrate that errors made during alignment may lead to biased estimates of divergence between sequences, but do not causally link the degree of MSA error with that bias. Each panel in figure 4 represents a different MSAM and shows the relationship between MSA error, characterized by average MSA distance from the true alignment, and divergence estimate error, taken to be the average sum of errors across individual branches in the phylogenetic tree for amino acid sequences and nucleotide sequences. As expected, as MSAs become more dissimilar to the true alignment, the error in branch length estimates becomes greater, although the nature of the relationship between these quantities depends on the MSAM used and whether one is examining amino acid or nucleotide sequences.

The degree of MSA error appears lower in amino acid sequences than nucleotide sequences. Under our nucleotide simulations with a tree length of 6.0 all MSAMs yield distances of around 0.9, which represents around 90% of pairwise homologies being inaccurately inferred (Blackburne and Whelan 2012). In contrast, for amino acid sequences simulated with a tree length of 6.0 the degree of error tends to range between 0.5 and 0.6, with the exception of ClustalW, which fares much worse. This difference may be attributable to the larger number of characters available in amino acid sequences. The larger number of characters leads to a reduced probability of back mutation to a character (e.g., A→R→A) and a lower probability of two similar strings of characters in a sequence occurring by chance. These two factors may allow longer runs of characters to be correctly aligned, reducing the tendency of underalignment and the underestimation of distances. Instead, these longer runs may result in the tendency to miss indels within the runs or to over extend the runs. Both these outcomes result in the alignment of nonhomologous and different characters, leading to the observed overestimation of distance.

Prank and FSA do not tend to follow this pattern since their MSAs have comparable distances from the true MSA as other MSAMs, but have lower distance errors. This difference is attributable to the types of error made. FSA, and to a lesser extent Prank, tend to have lower false positive (incorrectly assigned) pairwise homologies than other MSAMs (supplementary fig. S1, Supplementary Material online). This lower false positive rate means that there are fewer aligned residues and more gaps, leading to longer MSAs (supplementary fig. S2, Supplementary Material online). The MSAs produced by FSA, for example, are often many times longer than those produced by other programs. The lower false positive rate may result in lower divergence estimates because each false positive could require additional substitutions on a tree to explain it.

For similar degrees of divergence, defined by expected number of character replacements, MSAs from nucleotide sequences tend to be further from the true alignment (fig. 4). For nucleotides, all MSAMs show both very high false positive and false negatives (supplementary fig. S1, Supplementary Material online) Despite these extreme levels of MSA error, the overall levels of divergence error are lower than those of amino acid sequences. It is unclear how accurate divergence estimates can arise from mostly misaligned sequences, particularly for SATé, which has similar levels of error as other MSAMs but much more accurate divergence estimates. This may be because the iterative tree-search used to define the guide tree produces a more accurate tree for these divergent sequences than the k-mer methods used by other MSAMs (supplementary fig. S3, Supplementary Material online), which in turn leads to groups of true and false pairwise homologies that nevertheless reflect the history of the sequences.

### The Effect of Increasing the Number of OTUs on Inferred Sequence Divergence

The sections above all examine the effect of MSA on inferring divergence from eight OTUs, whereas most studies examine many more. In order to investigate the effect of increasing the number of OTUs on MSA and downstream analyses we also perform simulations on groups of 8, 64, and 512 OTUs where the tree and relative branch lengths shown in figure 1*A* is exactly embedded within the larger group of OTUs while spanning the entire tree topology. The MSA for this set of eight OTUs is extracted from the MSA obtained using all of the simulated sequences and analyzed in the same manner as those in figure 2. This experimental design allows the direct comparison between the results obtained here and those in the preceding part of this study. If an MSAM is unaffected by the number of OTUs then the evidence for bias in inferred tree length will remain the same regardless of the number of OTUs used in the full MSA.

**Fig. 5.—:**
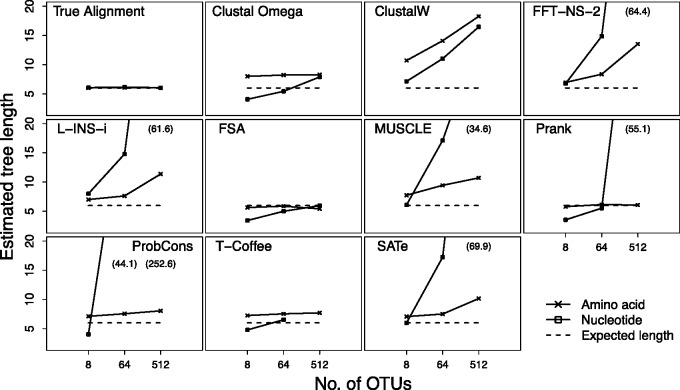
The effect of increasing the number of OTUs included during MSA on the tree length inferred from an identically structured subset of eight OTUs embedded within the full set of OTUs. The true total tree length of the eight OTUs, represented by the dotted line, is 6. Note that T-Coffee would not run successfully on nucleotide sequences from 512 OTUs.

Figure 5 shows that the number of OTUs included in the analysis affects the tree length inferred by many MSAMs. For amino acid sequences many of the MSAMs show a substantial increase in error of the tree length estimate, particularly for the 512 OTU-based data set. For example, ClustalW, MAFFT, MUSCLE, and SATé approximately double their error under the large 512 OTU MSA. Clustal Omega and T-Coffee maintain roughly the same degree of error for all numbers of OTUs, whereas Prank and FSA retain their accurate estimates of divergence across different numbers of OTUs. The inferred tree lengths from nucleotide sequences are generally much worse than those from amino acids. For 512 OTUs many MSAMs result in very long inferred tree lengths. The cause of these extreme estimates is evident when examining the extracted eight OTU MSAs, which consists mainly of gaps with very few aligned bases in each column. It may be more accurate to interpret these cases as the MSA step failing rather than the two-step process producing a heavily biased estimate.

### MSA Error and Phylogenetic Tree Estimation

Evidence for systematic error when estimating divergence affects many evolutionary analyses where relative branch length estimates are important, such as molecular dating or studying adaptation, but the most popular evolutionary analysis remains inferring a phylogenetic tree. Here, we use simulation to examine two related questions about the effect of MSA on phylogenetic tree inference.

### The Effect of MSA on Tree Estimates from Multifurcations

Previous studies have demonstrated that multifurcations (polytomies) can affect both ML and Bayesian inference, either through the strategies used to perform tree-search (Whelan and Money 2010) or through misspecification of the prior (Yang 2007a). To examine the effect of MSA on resolving multifurcations, we simulate data from an eight-taxa balanced tree where the root node is a four-way multifurcation (fig. 1*B*). Two of the subtrees attached to the multifurcation are short (root to tip length 0.12; each branch 0.06) and two are long (root to tip length 1.4; each branch 0.7), allowing us to investigate whether there is a bias toward one resolution of the multifurcation over the other two. If MSA induces no bias in the tree estimate then each tree will be chosen with frequency one-third and that frequency will be independent of the length of the sequences, demonstrated by the patterns shown by inference from the true alignment.

**Fig. 6.—:**
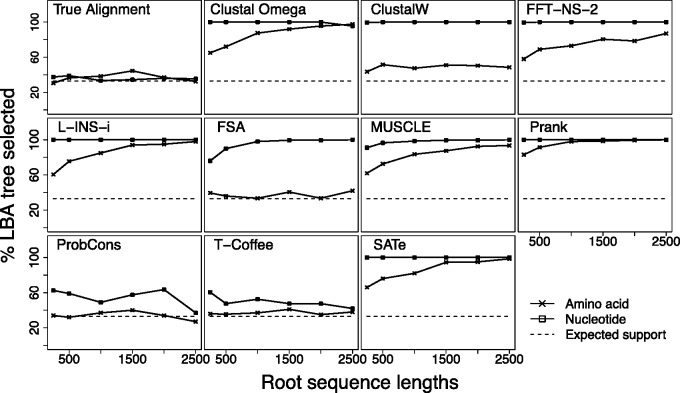
Frequency with which a polytomy is resolved to an LBA tree under ML for amino acid sequences and nucleotide sequences. Root sequence lengths are for amino acid sequences; nucleotide sequences have three times the length displayed in the *x* axis.

Figure 6 shows how frequently the multifurcation estimated to be the tree grouping the two long subtrees together under ML; a pattern reminiscent of an LBA artifact. The remaining fraction of the time, the tree is estimated to be one of the other two topologies with roughly even probability. For amino acid sequences all MSAMs that include a guide tree show a preference toward the LBA tree. The exceptions to this pattern are FSA, ProbCons, and T-Coffee, which incorporate consistency-based measures when scoring MSAs rather than just a guide tree based hierarchical alignment methods. The remaining MSAMs are more heavily reliant on a guide tree and more susceptible to LBA-type artifacts. Prank shows the strongest tendency to select the LBA tree, whereas the older ClustalW shows the weakest LBA artifact. Increasing the amount of sequence data causes MSAs produced by Clustal Omega, MAFFT, MUSCLE, Prank, and SATé to progressively tend toward the LBA tree, while the other MSAMs remain stable. For nucleotide sequences a similar, but more extreme pattern is observed. All of the MSAMs based on a guide tree show a very strong preference toward the tree that groups the two long subtrees together, even for very short sequences. The only MSAMs that does not exhibit this pattern are the consistency-based MSAMs T-Coffee and ProbCons. The effect of sequence length is less noticeable in nucleotide sequences since MSAs from most guide-tree-based MSAMs result in the LBA tree being estimated with very high frequency even for short root sequences.

### The Effect of MSA on Tree Support from Multifurcations

**Fig. 7.—:**
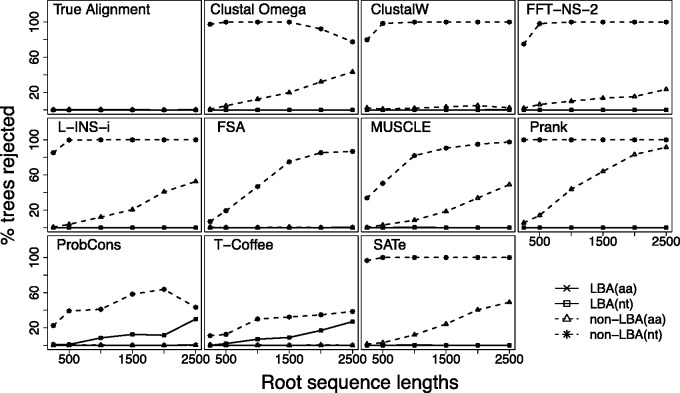
Frequency with which LBA and non-LBA trees are rejected using a RELL-based SH-test for amino acid and nucleotide sequences. Root sequence lengths are for amino acid sequences; nucleotide sequences have three times the length displayed in the *x* axis.

Demonstrating a tendency to resolve a multifurcation toward the LBA tree alone is a concern for phylogenetic studies. More important, however, is whether MSA can cause high levels of statistical support for that tree, leading to strong, but false, confidence in a particular resolution. Figure 7 shows the probability of each of the LBA and other resolutions of the multifurcation being rejected by the SH-test for amino acid and nucleotide sequences, respectively. Each panel in the figure represents results obtained from the MSAs of a specific aligner. These probabilities are obtained by counting how frequently trees are rejected in simulation (pSH < 0.05; full details in Materials and Methods). If the test is working correctly, then around 5% of trees will be rejected by chance. The results from the real alignments suggest that less than 5% of trees are rejected, indicating that the SH-test is conservative, in agreement with previous studies (Goldman et al. 2000).

Both sets of panels show there is a tendency for the SH-test to reject the two non-LBA trees and to not reject the LBA tree, although the strength of that tendency varies between MSAMs and the type of data analyzed. For nucleotide sequences, all MSAMs except MUSCLE, FSA, T-Coffee, and ProbCons tend to reject non-LBA trees for moderate and long root sequences. For amino acid sequences, as root sequence length increases the non-LBA trees are rejected increasingly frequently for all MSAMs, with the exception of FSA, ProbCons, T-Coffee, and to a lesser extent ClustalW. The error rates of programs appear related to two related properties of the MSAM: whether or not it uses a guide tree, and where it makes alignment errors on the tree.

We base our statement about the effect of the guide tree on two lines of evidence. The first is that progressive MSAMs that use a guide tree frequently demonstrate higher errors rates. MSAs obtained from Clustal Omega, the MAFFT algorithms, and Prank, for example, all show very strong tendencies to reject non-LBA trees for nucleotide sequences, and an increasing tendency to reject non-LBA trees for amino acid sequence as the root length increases. Iteratively improving the tree, even through the ML estimates in SATé, does not provide a noticeable improvement, suggesting that once the errors are established in the initial MSA they bias subsequent tree estimates to that tree. In contrast, the consistency-based ProbCons and the pseudostatistical MSAM FSA tend to show lower, but still worryingly high, error rates. The second line of evidence is several MSAMs show a very strong correlation between the choice of initial guide tree and bias in the tree estimate from the analysis of MSA. From the 200 simulations on our longest tree, Prank estimates an LBA guide tree 195 times, leading to rejection of non-LBA trees in 183/195 (93.8%) of those cases. This line of evidence does not hold for all MSAMs. Clustal Omega, for instance, estimates an LBA guide tree in only 24/200 simulations and still shows high rates of LBA type bias.

The effect of the placement of errors in the trees inferred from MSAs is evident from combining the information from figures 3^7^, and helps to explain the outlying error rates of some MSAMs. Figure 3 shows that tree estimates from FSA MSAs tend to underestimate the number of substitutions on internal branches, which could be expected to result in lower support for any tree, which could explain why it tends to reject very few trees for nucleotide sequence data and reject almost no trees for amino acid data. In contrast, figure 3 shows that Prank tends to overestimate the number of substitutions on internal branches, which may explain why it tends to have the highest rejection rate for non-LBA trees evident in amino acid MSAs. These placements of substitutions in the inferred trees may also be at least partly a product of the guide tree methods used, so should be viewed as a complementary explanation for high levels of erroneous non-LBA tree rejection.

Supplementary figure S4, Supplementary Material online, shows that bootstrap measures of tree support are also subject to these biases, suggesting the effects we observe are general and not limited to any individual topology-based test.

### LBA Artifacts Induced by MSA

The section above demonstrates that a multifurcation is preferentially resolved in favor of a bifurcating LBA-style tree. Note that our results do not demonstrate LBA in the usual manner, whereby sequence data generated from a Felsenstein-zone four taxa tree where a pair of long and short external branches are separated from one another by a short internal branch are falsely inferred by parsimony to come from a tree where the two long branches group together. Here, we extend those results using the pseudo-Felsenstein-zone tree shown in figure 1*C* with either 8 or 32 taxa falling into two long and two short subtrees separated by a single internal short branch. We examine the three rearrangements of those subtrees around this short internal branch, with the LBA-style tree being that which groups together the two long subtrees. Following the classic study by Huelsenbeck and Hillis (1993), we vary the length of the internal branch and the long and short subtrees, reporting how frequently the correct tree is inferred.

**Fig. 8.—:**
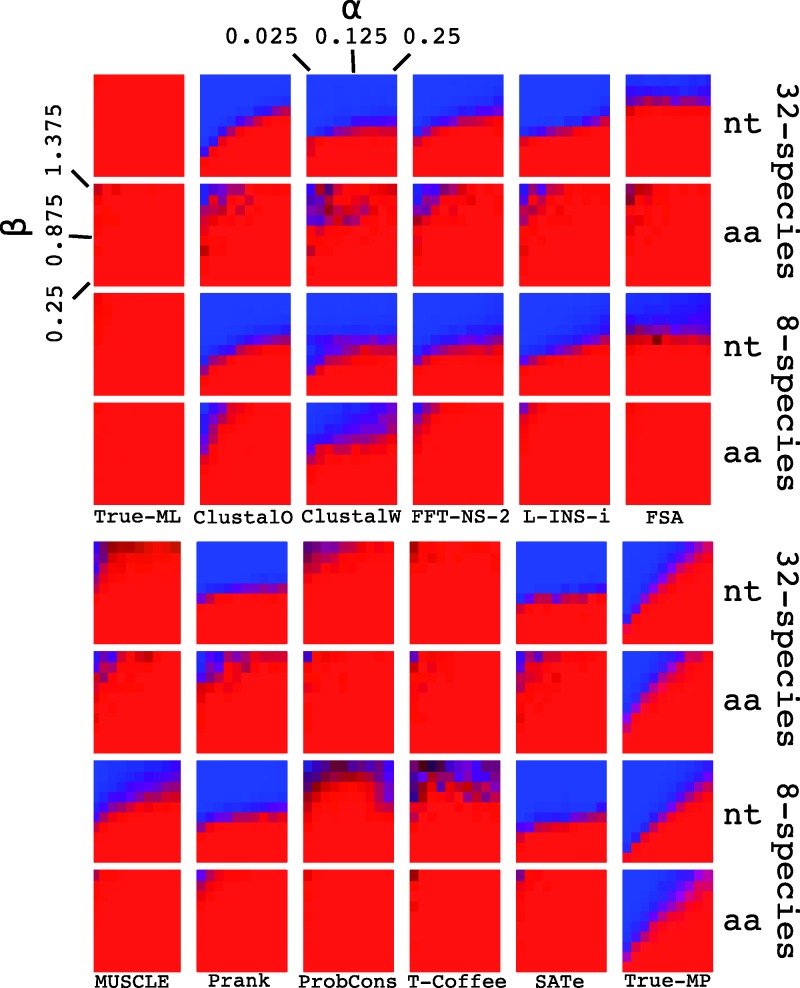
Heat maps showing the relative frequency that the correct tree (red squares) is estimated from data simulated under the Felsenstein-zone tree shown in figure 1*C* for data simulated under amino acid and nucleotide sequences. Blue colored squares represent the selection of an incorrect tree. For each panel, the *y* axis shows the long subtree length, labeled β in figure 1*C*, and takes values {0.25, 0.375, … , 1.375} and the *x* axis shows the short subtree length, labeled α in figure 1*C*, and takes values {0.025, 0.05, … , 0.25}. True-ML and True-MP show the frequency with which the true tree is estimated from the true (simulated) MSA using ML and MP, respectively.

Figure 8 shows tree estimates from data simulated under nucleotide and amino acid models. The left hand panel of the top four rows (True-ML) shows that, as expected from the property of statistical consistency, ML analyses based on the known alignments show absolute confidence for the true tree. The right hand panel of the four bottom rows shows tree estimates from MP, demonstrating the widely known LBA artifact and allowing us to compare the errors induced by MSA to this well-characterized problem. The intervening panels show the errors from ML tree inference based on the MSAs produced by different programs. The most striking result from figure 8 is that all MSAMs apart from the consistency-based ProbCons and to some extend T-Coffee demonstrate some degree of LBA artifact for nucleotide sequences, although the nature of that LBA artifact is somewhat different from that observed under MP. The LBA artifacts induced by MP tend to produce a clear diagonal line across the heat map, whereas those induced by MSA tend to be closer to a horizontal line. Moreover, moving from 8- to 32-taxa simulations appears to lessen the severity of the MP LBA artifact, which is probably attributable to increased taxon sampling and shorter average branch lengths in the subtrees. In contrast, for the majority of MSAMs there is little difference between the two numbers of taxa. The exceptions to this are MUSCLE and FSA, which both show a noticeable improvement under 32-taxa simulations. These two observations suggest there is a divergence threshold for nucleotide data, after which it becomes very difficult to infer accurate guide trees and MSAs.

The amino acid sequences show a much-improved scenario. Although MP causes a clear LBA artifact, for eight-taxa simulations none of the MSAMs show strong evidence of LBA artifacts, except under the most extreme conditions (top left of heat maps; very long and very short subtrees). This pattern is maintained for the 32-taxa simulations, with the exception of ClustalW that begins to show a pattern reminiscent of the MP LBA artifact. This observation suggests that the older ClustalW MSAM tends to make more errors as additional sequences are added to the tree even if they increase taxon sampling. These errors may include incorrectly carrying gap information up the tree during MSA, which has been addressed by iterative MSA improvement (Edgar 2004) and phylogenetic gap placement (Löytynoja and Goldman 2008) in more modern MSAMs

### Perspectives and Recommendations

This simulation study shows that even correctly specified phylogenetic methods are susceptible to a range of biases introduced by the MSA step. These results underscore the important fact that the consistency proofs associated for statistical methods of phylogenetic and phylogenomic inference are not generally applicable to real-world analysis because they all assume that sequence evolution occurs only through substitution and that homology relationships between characters are known with certainty. In reality, sequence evolution proceeds through substitution, insertion, deletion, and many more processes. The MSA step attempts to capture all of these complex processes when assigning sitewise homology, allowing phylogenetic models to describe each column’s evolution solely through substitution. The inability for MSA to capture this complexity leads to errors in downstream inference at two interconnected levels: the inference of phylogenetic trees and the estimates of branches upon those trees.

### Perspectives on Studies Requiring a Tree Topology

Our study suggests that MSA can affect phylogenetic tree estimates leading to systematic bias similar to LBA artifacts observed for MP. These biases are strongest in the resolution of multifurcations in relatively divergent nucleotide sequences, but also evident in the resolution of multifurcations in amino acid sequences. These errors come from two compounding sources. First is the influence of the chosen guide tree, which can be considered the model that MSAMs follow and many MSAMs tend to overfit that model. This overfitting may manifest itself in the resultant alignment by inferring homologies between residues that do not share a common ancestor, but fit the guide tree (false positives). Alternatively, removing homology relationships that contradict the guide tree, but do represent shared descent, may also cause overfitting (false negatives). There are no tools available to readily identify these types of false positive and false negative, but given our observations it is reasonable to infer that these effects lead to MSAs where substitutions too closely follow the guide tree and result in biased topology estimates.

The second potential source of error comes from the relatively simple clustering algorithms used to construct guide trees in MSAMs. These algorithms are often based on simple similarity measures, rather than evolutionary distances, which are known to be susceptible to LBA and may lead to such artifacts in guide trees. It is the systematic bias toward recovering LBA guide trees, coupled with overfitting the MSA to those LBA guide trees, that is the probable cause of the LBA-type artifacts that we observe in the downstream evolutionary inference. Improved guide trees will not correct for the overfitting of MSAs, which is expected to lead to overconfidence in whichever guide tree topology is selected, but it could reduce the bias toward specific types of topology, such as LBA artifacts.

We observe that amino acids help alleviate this problem, removing LBA artifacts from Felsenstein-zone trees and substantially reducing biases when resolving multifurcations. The performance of amino acid sequences relative to nucleotide sequences may result from them being more robust to overfitting and guide tree error. The larger number of character states in amino acid sequence means it may be easier to differentiate true and false homology inferences. Back mutation is less likely, reducing false homology calls, and the larger possible sequence-space may allow easier identification of homologous regions. These factors also make it easier to infer guide trees from amino acid sequences using similarity-based methods such as k-mer clustering, leading to less biased guide-trees and a reduction in LBA-type artifacts. These factors suggest these are no reasonable grounds to perform MSA on protein coding nucleotide sequences. Even when one requires a nucleotide alignment, the approach of translation, MSA, followed by back translation seems likely to yield better results.

The use of amino acid sequences does not remove biased resolution of multifurcations during MSA, so we suggest some practical precautions when analysing such data. Strong statistical support for very short internal branches should be treated with caution, especially for divergent sequences where the MSA appears uncertain. This suggestion can be practically assessed by examining the MSA and the point estimate and statistical support for trees under a range of different MSAMs. The MSAMs should minimally include an evolution-MSAM and a similarity-MSAM (Blackburne and Whelan 2013) and preferably both progressive and consistency similarity-MSAMs should be examined. The degree of disagreement between the resultant MSAs can be assessed using a pairwise distance matrix produced using (e.g.,) MetAl distances (Blackburne and Whelan 2012). Disagreement between trees and their statistical support can be assessed in a similar manner using (e.g.,) Robinson–Fould distances or *P* values. We note that no evidence of disagreement is only a necessary condition for trust in a tree, and that the tree estimate may still be inaccurate even when there is no obvious disagreement between the MSA and tree-based statistics.

The recently published study by Karin et al. (2014) provides an alternative approach based on the long-standing practice of MSA filtering. The study shows that filtering may reduce, although not eliminate, SH-test artifacts caused by alignment. They use a GUIDANCE-based approach (Penn et al. 2010), which varies the guide tree of MAFFT to identify regions of the MSA susceptible to error from the guide tree. The GUIDANCE approach is a significant improvement over simpler filtering strategies such as GBlocks (Castresana 2000), but may need to be combined with improvements to MSAMs and some of the suggestions above to reduce the potential biases caused by alignment.

### The Outlook for Studies Requiring Divergence Estimates

Our results suggest that MSA uncertainty leads to biased estimates of sequence divergence throughout an evolutionary tree, and those biases are dependent on the MSAM used, the number of OTUs included in a study, and the type of data analyzed. In common with tree estimation, our results suggest estimates of branch lengths based on nucleotide sequence MSAs are particularly difficult to estimate, even for relatively closely related sequences. The outlook for branch length estimates based on amino acid sequences is better for closely related sequences, but the accuracy of estimates remains low and unpredictable for more divergent sequences. Prank tends to have to most stable estimates as sequences diverge, but there is a notable tendency to underestimation of external branches and overestimation of the root branch for the most divergent sequences. Our study of divergence estimates uses a balanced tree with equal branch lengths for simulation, so errors introduce by an incorrect guide tree have a more limited impact on the systematic errors we observe. For the majority of MSAMs the observed errors may be a consequence of overfitting the guide tree, even for the consistency-based MSAM ProbCons, which uses a guide tree to obtain an initial MSA. The exception to this is FSA, which instead uses a sequence annealing approach to obtain an MSA, and may be reflected in the unusual tendency of its MSAs to infer too few changes in the internal branches and too many changes on the external branches. Our results also show for many MSAMs there is a clear negative effect from including additional sequences in the two-step process. Although this study concentrates on relatively small data sets where the error is easier to quantify, these results suggest the errors caused by MSA may be even more prevalent in larger data sets.

The wide range of biases in branch length estimates caused by MSA has the potential to affect all areas of evolutionary inference that rely on the accuracy of those estimates. For example, previous studies characterizing the effect of MSA on dN/dS-based tests of adaptive evolution (Fletcher and Yang 2010; Markova-Raina and Petrov 2011) can be interpreted in the light of our study as differential errors being made in dN and dS distance estimates. Other areas that rely on accurate and unbiased estimates of divergence are also likely to be affected, including methods of molecular dating (Welch and Bromham 2005); models of species divergence (Stadler 2011); methods for inferring patterns of temporal and spatial heterogeneity (Wang et al. 2007; Whelan 2008; Whelan et al. 2011); and inference of protein secondary structure using mixture models (Le and Gascuel 2010) or hidden Markov models (Thorne et al. 1996). Further study is required to characterize the degree of errors induced by MSA for these types of studies.

To illustrate how these apparent biases can affect analyses we shall discuss their potential effects on the currently popular field of estimating diversification rates from molecular data (Rabosky and McCune 2010). Widely used tools in this area include the BAMM package (Rabosky 2014) and the GEIGER package (Harmon et al. 2008; Alfaro et al. 2009). These methods use model-based approaches to infer shifts in diversification rates from ultrametric phylogenetic trees and their branch lengths. Our results suggest that the MSA step can affect the branch lengths of those trees in way that may systematically bias diversification estimates. If the tree were based on amino acid sequences, for example, using FSA would tend to underestimate deep branches resulting in greater diversification rates towards the base of the tree. In contrast, using SATé may lead to overestimation of root branches, which may appear as evidence for decreased diversification rates toward the base of the tree. Given our results from larger data sets, it also seems reasonable to presume that denser sampling in some clades may affect their branch length estimates, particularly for nucleotide sequences obtained from intergenic markers.

We can offer a few recommendations for studies requiring the accurate estimation of branch lengths beyond the recommendations we make for tree-based analyses. Using amino acid based MSAs and comparing between evolutionary and similarity-based MSAs may reduce error, and advanced filtering methods such as GUIDANCE may also help, particularly for divergent sequences where k-mer derived guide trees may become unstable. Recent work examining the performance of codon-based tests of natural selection may provide an alternative solution. Simulations have shown that statistical alignment methods can erase errors in dN/dS estimates (Redelings 2014); a type of error closely related to that of divergence estimate error. The problem is that these methods come at great computational, with single analyses on small data sets taking many hours, preventing their general application to genome-scale studies. They do, however, suggest that faster and more widely applicable MSAMs based on the principles of statistical alignment may provide the key to reducing errors in divergence estimates.

## Supplementary Material

Supplementary figures S1–S4 are available at *Genome Biology and Evolution* online (http://www.gbe.oxfordjournals.org/).

Supplementary Data

## References

[evv127-B1] Alfaro ME 2009 Nine exceptional radiations plus high turnover explain species diversity in jawed vertebrates. Proc Natl Acad Sci U S A. 106:13410–13414. 19633192 10.1073/pnas.0811087106 PMC2715324

[evv127-B2] Anisimova M Cannarozzi G Liberles DA 2010 Finding the balance between the mathematical and biological optima in multiple sequence alignment. Trends Evol Biol. 2:e7.

[evv127-B3] Arbiza L Patricio M Dopazo H Posada D 2011 Genome-wide heterogeneity of nucleotide substitution model fit. Genome Biol Evol. 3:896. 21824869 10.1093/gbe/evr080 PMC3175760

[evv127-B4] Arunapuram P 2013 StatAlign 2.0: combining statistical alignment with RNA secondary structure prediction. Bioinformatics 29:654–655. 23335014 10.1093/bioinformatics/btt025

[evv127-B5] Blackburne BP Whelan S 2012 Measuring the distance between multiple sequence alignments. Bioinformatics 28:495–502. 22199391 10.1093/bioinformatics/btr701

[evv127-B6] Blackburne BP Whelan S 2013 Class of multiple sequence alignment algorithm affects genomic analysis. Mol Biol Evol. 30:642–653. 23144040 10.1093/molbev/mss256

[evv127-B7] Blackshields G Wallace IM Larkin M Higgins DG 2006 Analysis and comparison of benchmarks for multiple sequence alignment. In Silico Biol. 6:321–339. 16922695

[evv127-B8] Bradley RK 2009 Fast statistical alignment. PLoS Comput Biol. 5:e1000392. 19478997 10.1371/journal.pcbi.1000392 PMC2684580

[evv127-B9] Castresana J 2000 Selection of conserved blocks from multiple alignments for their use in phylogenetic analysis. Mol Biol Evol. 17:540–552. 10742046 10.1093/oxfordjournals.molbev.a026334

[evv127-B10] Cooper GM 2004 Characterization of evolutionary rates and constraints in three mammalian genomes. Genome Res. 14:539–548. 15059994 10.1101/gr.2034704 PMC383297

[evv127-B11] Dayhoff M Schwartz R Orcutt B 1978 A model of evolutionary change in proteins. In: Dayhoff M, editor. Atlas of protein sequence and structure. Washington, D.C.: National Biomedical Research Foundation vol. 5, p. 345–352.

[evv127-B12] Do CB Mahabhashyam MS Brudno M Batzoglou S 2005 ProbCons: probabilistic consistency-based multiple sequence alignment. Genome Res. 15:330–340. 15687296 10.1101/gr.2821705 PMC546535

[evv127-B13] Edgar RC 2004 MUSCLE: multiple sequence alignment with high accuracy and high throughput. Nucleic Acids Res. 32:1792–1797. 15034147 10.1093/nar/gkh340 PMC390337

[evv127-B14] Felsenstein J 1978 Cases in which parsimony or compatibility methods will be positively misleading. Syst Zool. 27:401–410.

[evv127-B15] Felsenstein J 2003 Inferring phylogenies. Sunderland (MA): Sinauer Associates.

[evv127-B16] Fletcher W Yang Z 2009 INDELible: a flexible simulator of biological sequence evolution. Mol Biol Evol. 26:1879–1888. 19423664 10.1093/molbev/msp098 PMC2712615

[evv127-B17] Fletcher W Yang Z 2010 The effect of insertions, deletions, and alignment errors on the branch-site test of positive selection. Mol Biol Evol. 27:2257–2267. 20447933 10.1093/molbev/msq115

[evv127-B18] Galtier N Gouy M 1998 Inferring pattern and process: maximum-likelihood implementation of a nonhomogeneous model of DNA sequence evolution for phylogenetic analysis. Mol Biol Evol. 15:871–879. 9656487 10.1093/oxfordjournals.molbev.a025991

[evv127-B19] Goldman N Anderson JP Rodrigo AG 2000 Likelihood-based tests of topologies in phylogenetics. Syst Biol. 49:652–670. 12116432 10.1080/106351500750049752

[evv127-B20] Harmon LJ Weir JT Brock CD Glor RE Challenger W 2008 GEIGER: investigating evolutionary radiations. Bioinformatics 24:129–131. 18006550 10.1093/bioinformatics/btm538

[evv127-B21] Huelsenbeck JP Hillis DM 1993 Success of phylogenetic methods in the four-taxon case. Syst Biol. 42:247–264.

[evv127-B22] Karin EL Susko E Pupko T 2014 Alignment errors strongly impact likelihood-based tests for comparing topologies. Mol Biol Evol. 31:3057–3067. 25085999 10.1093/molbev/msu231

[evv127-B23] Katoh K Standley DM 2013 MAFFT multiple sequence alignment software version 7: improvements in performance and usability. Mol Biol Evol. 30:772–780. 23329690 10.1093/molbev/mst010 PMC3603318

[evv127-B24] Kosiol C Bofkin L Whelan S 2006 Phylogenetics by likelihood: evolutionary modeling as a tool for understanding the genome. J Biomed Inform. 39:51–61. 16226061 10.1016/j.jbi.2005.08.003

[evv127-B25] Le SQ Gascuel O 2010 Accounting for solvent accessibility and secondary structure in protein phylogenetics is clearly beneficial. Syst Biol. 59:277–287. 20525635 10.1093/sysbio/syq002

[evv127-B26] Liberles DA 2012 The interface of protein structure, protein biophysics, and molecular evolution. Protein Sci. 21:769–785. 22528593 10.1002/pro.2071 PMC3403413

[evv127-B27] Liu K 2012 SATe-II: very fast and accurate simultaneous estimation of multiple sequence alignments and phylogenetic trees. Syst Biol. 61:90–106. 22139466 10.1093/sysbio/syr095

[evv127-B28] Liu K Raghavan S Nelesen S Linder CR Warnow T 2009 Rapid and accurate large-scale coestimation of sequence alignments and phylogenetic trees. Science 324:1561–1564. 19541996 10.1126/science.1171243

[evv127-B29] Löytynoja A Goldman N 2008 Phylogeny-aware gap placement prevents errors in sequence alignment and evolutionary analysis. Science 320:1632–1635. 18566285 10.1126/science.1158395

[evv127-B30] Markova-Raina P Petrov D 2011 High sensitivity to aligner and high rate of false positives in the estimates of positive selection in the 12 *Drosophila* genomes. Genome Res. 21:863–874. 21393387 10.1101/gr.115949.110 PMC3106319

[evv127-B31] Mirarab S Warnow T 2011 FastSP: linear time calculation of alignment accuracy. Bioinformatics 27:3250–3258. 21984754 10.1093/bioinformatics/btr553

[evv127-B32] Morrison DA Ellis JT 1997 Effects of nucleotide sequence alignment on phylogeny estimation: a case study of 18S rDNAs of Apicomplexa. Mol Biol Evol. 14:428–441. 9100373 10.1093/oxfordjournals.molbev.a025779

[evv127-B33] Notredame C Higgins DG Heringa J 2000 T-Coffee: a novel method for fast and accurate multiple sequence alignment. J Mol Biol. 302:205–218. 10964570 10.1006/jmbi.2000.4042

[evv127-B34] Penn O 2010 GUIDANCE: a web server for assessing alignment confidence scores. Nucleic Acids Res. 38:W23–W28. 20497997 10.1093/nar/gkq443 PMC2896199

[evv127-B35] Rabosky DL 2014 Automatic detection of key innovations, rate shifts, and diversity-dependence on phylogenetic trees. PLoS One 9:e89543. 24586858 10.1371/journal.pone.0089543 PMC3935878

[evv127-B36] Rabosky DL McCune AR 2010 Reinventing species selection with molecular phylogenies. Trends Ecol Evol. 25:68–74. 19740566 10.1016/j.tree.2009.07.002

[evv127-B37] Redelings B 2014 Erasing errors due to alignment ambiguity when estimating positive selection. Mol Biol Evol. 31:1979–1993. 24866534 10.1093/molbev/msu174 PMC4155473

[evv127-B38] Redelings B Suchard M 2005 Joint Bayesian estimation of alignment and phylogeny. Syst Biol. 54:401–418. 16012107 10.1080/10635150590947041

[evv127-B39] Rogers JS 1997 On the consistency of maximum likelihood estimation of phylogenetic trees from nucleotide sequences. Syst Biol. 46:354–357. 11975346 10.1093/sysbio/46.2.354

[evv127-B40] Sankoff D Kruskal J. 1983 Time warps, string edits, and macromolecules: the theory and practice of sequence comparison. Reading (MA): Addison-Wesley.

[evv127-B41] Shimodaira H Hasegawa M 1999 Multiple comparisons of log-likelihoods with applications to phylogenetic inference. Mol Biol Evol. 16:1114–1116.

[evv127-B42] Sievers F 2011 Fast, scalable generation of high-quality protein multiple sequence alignments using Clustal Omega. Mol Syst Biol. 7:539. 21988835 10.1038/msb.2011.75 PMC3261699

[evv127-B43] Stadler T 2011 Mammalian phylogeny reveals recent diversification rate shifts. Proc Natl Acad Sci U S A. 108:6187–6192. 21444816 10.1073/pnas.1016876108 PMC3076834

[evv127-B44] Thompson JD Higgins DG Gibson TJ 1994 CLUSTAL W: improving the sensitivity of progressive multiple sequence alignment through sequence weighting, position-specific gap penalties and weight matrix choice. Nucleic Acids Res. 22:4673–4680. 7984417 10.1093/nar/22.22.4673 PMC308517

[evv127-B45] Thorne J Kishino H Felsenstein J 1992 Inching toward reality: an improved likelihood model of sequence evolution. J Mol Evol. 34:3–16. 1556741 10.1007/BF00163848

[evv127-B46] Thorne JL Goldman N Jones DT 1996 Combining protein evolution and secondary structure. Mol Biol Evol. 13:666–673. 8676741 10.1093/oxfordjournals.molbev.a025627

[evv127-B47] Thorne JL Kishino H Felsenstein J 1991 An evolutionary model for maximum likelihood alignment of DNA sequences. J Mol Evol. 33:114–124. 1920447 10.1007/BF02193625

[evv127-B48] Wallace IM O'Sullivan O Higgins DG Notredame C 2006 M-Coffee: combining multiple sequence alignment methods with T-Coffee. Nucleic Acids Res. 34:1692–1699. 16556910 10.1093/nar/gkl091 PMC1410914

[evv127-B49] Wang HC Spencer M Susko E Roger AJ 2007 Testing for covarion-like evolution in protein sequences. Mol Biol Evol. 24:294–305. 17056642 10.1093/molbev/msl155

[evv127-B50] Welch JJ Bromham L 2005 Molecular dating when rates vary. Trends Ecol Evol. 20:320–327. 16701388 10.1016/j.tree.2005.02.007

[evv127-B51] Whelan S 2008 Spatial and temporal heterogeneity in nucleotide sequence evolution. Mol Biol Evol. 25:1683–1694. 18502771 10.1093/molbev/msn119

[evv127-B52] Whelan S Blackburne BP Spencer M 2011 Phylogenetic substitution models for detecting heterotachy during plastid evolution. Mol Biol Evol. 28:449–458. 20724379 10.1093/molbev/msq215

[evv127-B53] Whelan S de Bakker PIW Goldman N 2003 Pandit: a database of protein and associated nucleotide domains with inferred trees. Bioinformatics 19:1556–1563. 12912837 10.1093/bioinformatics/btg188

[evv127-B54] Whelan S de Bakker PIW Quevillon E Rodriguez N Goldman N 2006 PANDIT: an evolution-centric database of protein and associated nucleotide domains with inferred trees. Nucleic Acids Res. 34:D327–D331. 16381879 10.1093/nar/gkj087 PMC1347450

[evv127-B55] Whelan S Goldman N 2001 A general empirical model of protein evolution derived from multiple protein families using a maximum-likelihood approach. Mol Biol Evol. 18:691–699. 11319253 10.1093/oxfordjournals.molbev.a003851

[evv127-B56] Whelan S Money D 2010 The prevalence of multifurcations in tree-space and their implications for tree-search. Mol Biol Evol. 27:2674–2677. 20584772 10.1093/molbev/msq163

[evv127-B57] Wong K Suchard M Huelsenbeck J 2008 Alignment uncertainty and genomic analysis. Science 319:473–476. 18218900 10.1126/science.1151532

[evv127-B58] Yang ZH 1994 Estimating the pattern of nucleotide substitution. J Mol Evol. 39:105–111. 8064867 10.1007/BF00178256

[evv127-B59] Yang ZH 2006 Computational molecular evolution. Oxford:Oxford University Press.

[evv127-B60] Yang ZH 2007a Fair-balance paradox, star-tree paradox, and Bayesian phylogenetics. Mol Biol Evol. 24:1639–1655. 17488737 10.1093/molbev/msm081

[evv127-B61] Yang ZH 2007b PAML 4: phylogenetic analysis by maximum likelihood. Mol Biol Evol. 24:1586–1591 17483113 10.1093/molbev/msm088

