# Supplementary material for: Evidence of Statistical Inconsistency of Phylogenetic Methods in the Presence of Multiple Sequence Alignment Uncertainty
Source: Genome Biol Evol. 2015 Jul 1;7(8):2102–16. doi: 10.1093/gbe/evv127 (PMC4558847; doi:10.1093/gbe/evv127)
Supplement: Supplementary Data [file supp_7_8_2102__index.html]

Evidence of Statistical Inconsistency of Phylogenetic Methods in the Presence of Multiple Sequence Alignment Uncertainty — Supplementary Data 

# Evidence of Statistical Inconsistency of Phylogenetic Methods in the Presence of Multiple Sequence Alignment Uncertainty

## Supplementary Data

files

- Supplementary Data - pdf file
