# Supplementary material for: Evidence of Statistical Inconsistency of Phylogenetic Methods in the Presence of Multiple Sequence Alignment Uncertainty
Source: Genome Biol Evol. 2015 Jul 1;7(8):2102–16. doi: 10.1093/gbe/evv127 (PMC4558847; doi:10.1093/gbe/evv127)
Supplement: Supplementary Data [file supp_evv127_Hossain_et_al_2015_SuppMat.pdf]

Supplementary Figure 1

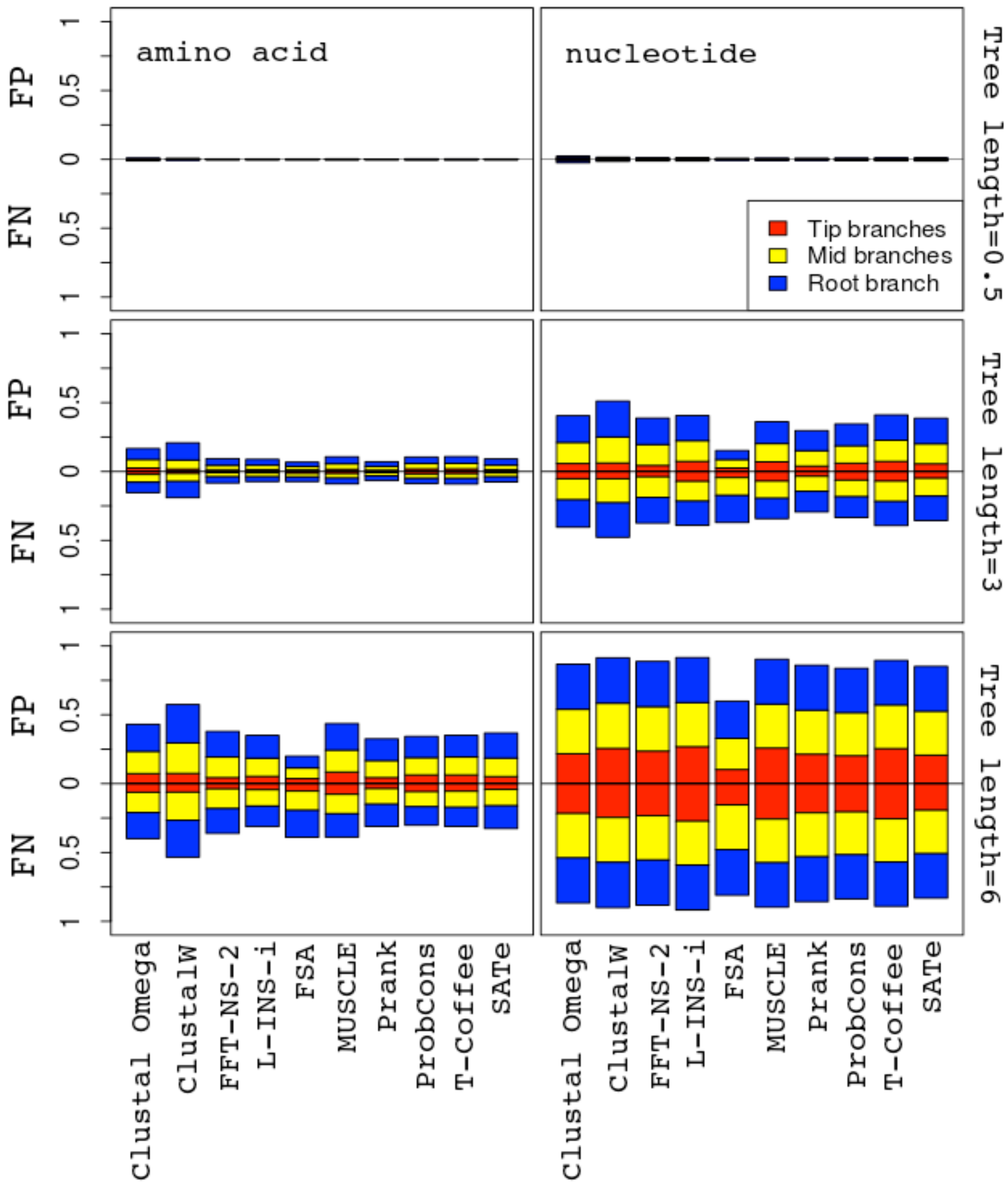

Supplementary Figure 1. As longer (more divergent) trees are used to simulate sequences, alignment programs increasingly fail to establish correct homology among sequences. More and more non-homologous residues are placed together (false positives; FP) as well as homologous residues are placed in different sites placing gaps (false negative; FN). The three left panels contains stacked bar plots representing average FP and FN values across the tip, mid and top branches (as in Figure 1A) for amino acid sequences while the right panels represent nucleotide sequences. Top, middle and the bottom rows display sequences simulated from tree lengths 0.5, 3 and 6 respectively.

Supplementary Figure 2

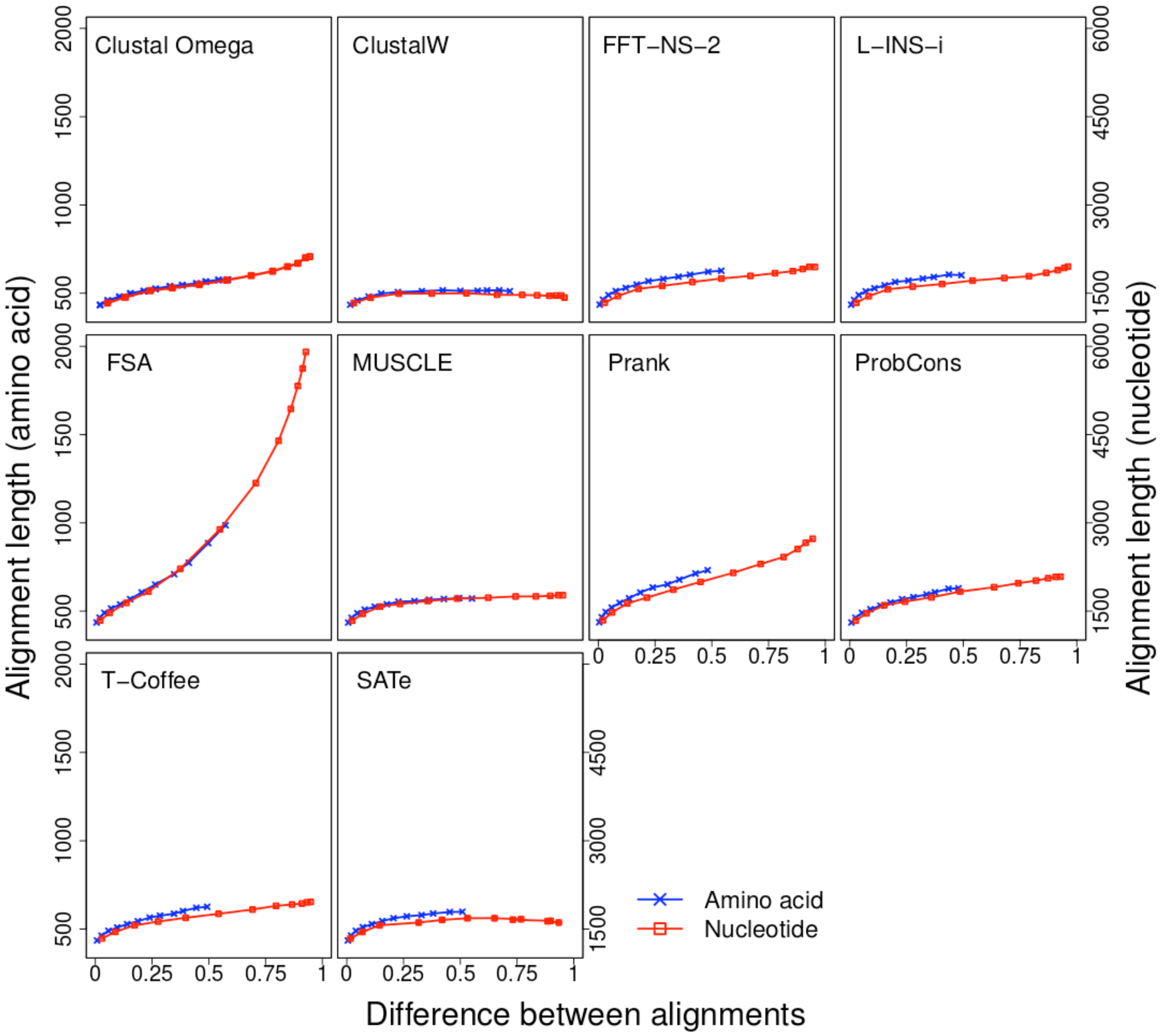

Supplementary Figure 2. Multiple sequence alignments are generated from sets of sequences simulated from 408bp (1224bp) long root amino acid (nucleotide) sequences with tree lengths={0.5, 1, 1.5, 2, 2.5, 3, 3.5, 4, 4.5, 5, 5.5, 6}. MSAs from different aligners are then compared with the true alignment using MetAl's d_{evol} metric to calculate MSA errors. MSAs got more and more different than their respective true alignments with increasing tree lengths (divergence) used for simulating those sequences. Alignments are longer for more divergent sequences except for ClustalW alignments; FSA alignments are the longest for both amino acid and nucleotide alignments.

Supplementary Figure 3

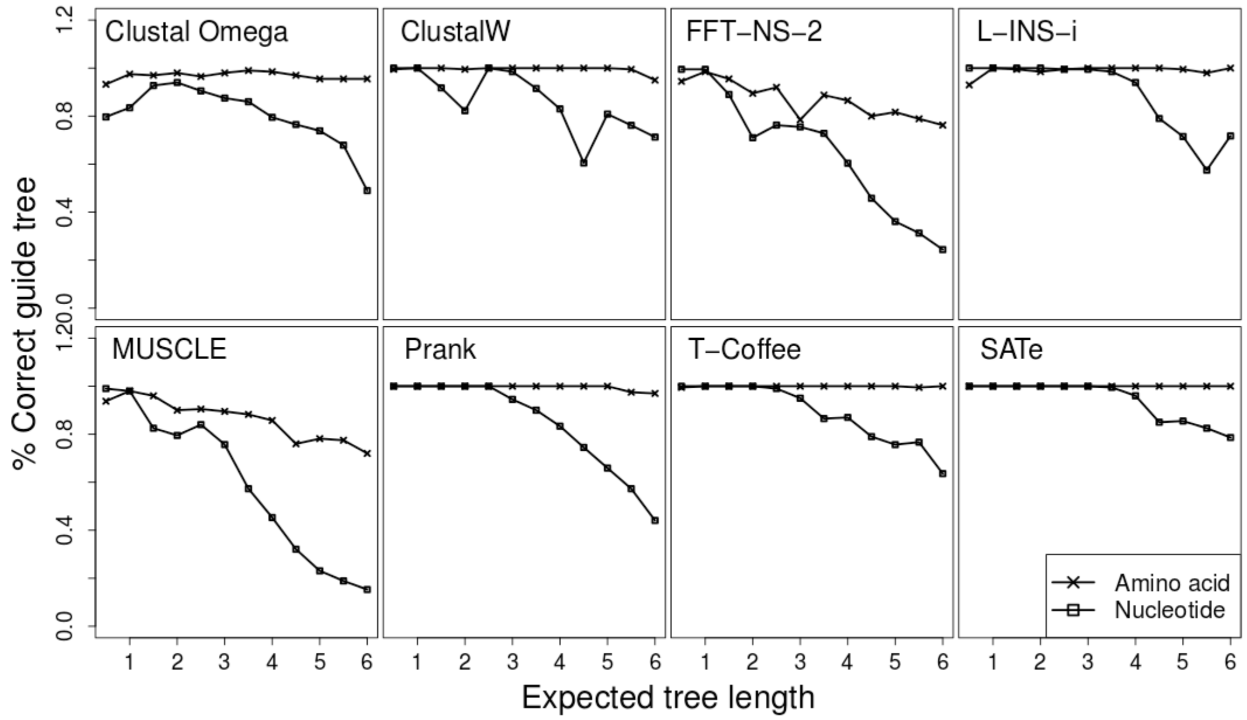

Supplementary Figure 3. Multiple sequence alignment programs (MSAMs) apart from FFT-NS-2 and MUSCLE produced mostly correct guide trees for aligning amino acid sequences irrespective of the tree lengths used for simulating those sequences. In contrast, less and less number of correct guide trees were produced by the MSAMs during the alignment of more divergent nucleotide sequences. The sequences were simulated using the phylogenetic tree shown in Figure 1A.

Supplementary Figure 4

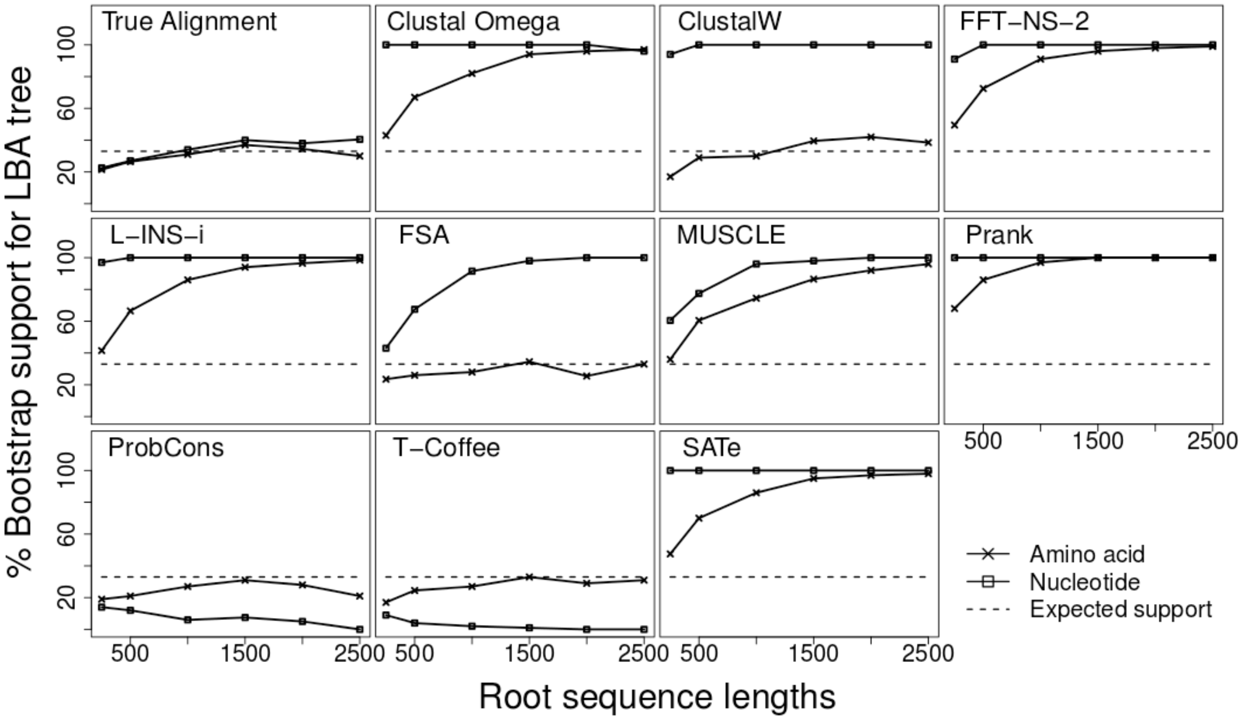

Supplementary Figure 4. Phylogenetic trees inferred from the MSAs produced by most of the aligners show strong bias towards LBA tree evident from the bootstrap supports of it while we expect all three trees to have similar bootstrap support equal to 33%. Bootstrap support of the LBA tree increases with the increase of root sequence length for amino acid alignments for most aligners. FSA, ProbCons and T-Coffee amino acid alignments show an expected bootstrap support close to 33% for the LBA tree. Bootstrap support for the LBA tree reaches 100% even for shorter nucleotide sequence alignments for most aligners. Only ProbCons and T-Coffee nucleotide alignments show strong (near 100%) bootstrap support for non-LBA trees.
